# Diagnostic uncertainty and management of low-grade appendiceal mucinous neoplasm—a case report and review of the literature

**DOI:** 10.1093/jscr/rjae717

**Published:** 2024-11-18

**Authors:** Charles Lu, Veysel K Embel, Mackenzie E Fox, Robin Donne, Glenn S Parker

**Affiliations:** Department of Surgery, Hackensack Meridian Health Jersey Shore University Medical Center, Neptune, NJ 07756, United States; Department of Surgery, Hackensack Meridian Health Jersey Shore University Medical Center, Neptune, NJ 07756, United States; Department of Surgery, Hackensack Meridian Health Jersey Shore University Medical Center, Neptune, NJ 07756, United States; Department of Surgery, Hackensack Meridian Health Jersey Shore University Medical Center, Neptune, NJ 07756, United States; Department of Surgery, Hackensack Meridian Health Jersey Shore University Medical Center, Neptune, NJ 07756, United States

**Keywords:** appendiceal mucinous neoplasm, hemicolectomy, appendectomy, mucocele, mucin, pseudomyxoma peritonei

## Abstract

Low-grade appendiceal mucinous neoplasm (LAMN) is a rare entity identified in ~1% of patients undergoing appendectomy. The presentation often varies, making diagnosis challenging. Timely identification and treatment are critical to prevent rupture, which may lead to pseudomyxoma peritonei. We describe the case of a 41-year-old male who presented for evaluation of acute right lower quadrant abdominal pain. The clinical impression was consistent with appendicitis with a clinical suspicion for underlying malignancy. The patient was brought to the operating room for an exploratory laparotomy and right hemicolectomy, revealing low-grade appendiceal mucinous neoplasm. The diagnosis of low-grade appendiceal mucinous neoplasm can be challenging given the variable presentation and imaging findings. Early recognition and treatment are imperative to prevent progression to pseudomyxoma peritonei. Our case report seeks to contribute to the ongoing literature and provide a review of the current knowledge.

## Introduction

**Figure 1:**
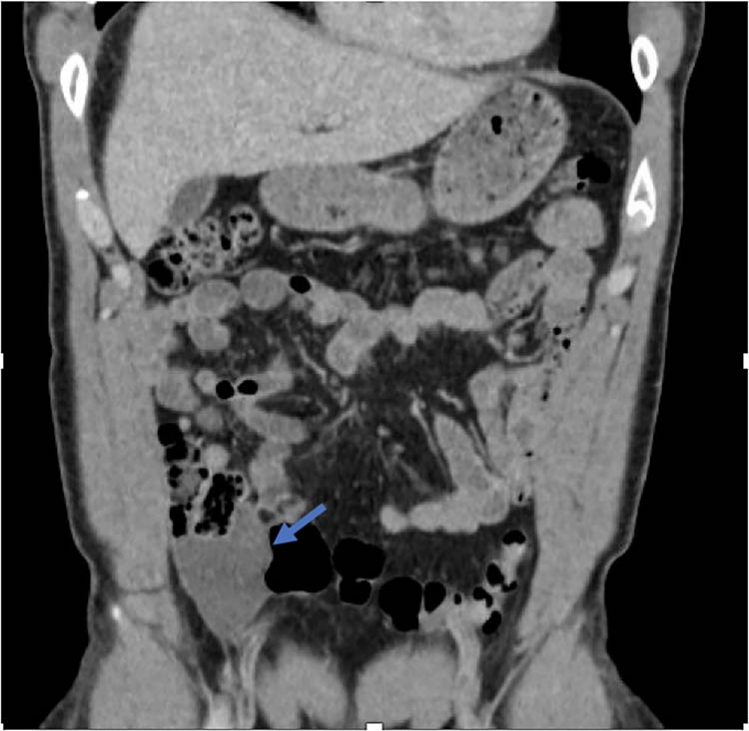
Fluid collection is present in the expected location of the appendix that measures 5.2 × 2.9 × 6.6 cm (blue arrow). The appendix is not seen as a separate and distinct structure.

Low-grade appendiceal mucinous neoplasm (LAMN) is a rare entity present in ~1% of appendectomies [1, 2]. It is described by the World Health Organization (WHO) as one of three main categories of mucinous neoplasms: mucinous adenoma, LAMN, and appendiceal adenocarcinoma. The features of LAMN include acellular or cellular extra-appendiceal mucin, and there is a predisposition for female patients over 50 years of age [2–4]. If left untreated, sequelae include rupture of the appendix and mucocele with potential progression to pseudomyxoma peritonei. This is associated with high morbidity and mortality [1–4]. The clinical presentation of LAMN can be quite variable. Some patients are asymptomatic with the lesion incidentally identified during imaging or other operative procedures, while other patients may present with abdominal pain, weight loss, and acute appendicitis. In advanced disease, there may be the presence of pseudomyxoma peritonei, abdominal distension, and abdominal hernia [5–7]. Thus, imaging is an important tool in diagnosis and management. Computerized tomography (CT) is particularly useful when identifying legions in the ileocecal region, as it offers multi-planar, high-definition anatomic evaluation of the intra-abdominal contents [8]. LAMN, if ruptured, may appear on CT as fluid collections with a similar density to water in the absence of visualization of the appendix as a separate structure or evidence of active inflammation (Fig. 1). In this report, we describe a case of LAMN initially presumed to be appendicitis without the classic of an acute inflammatory process ultimately treated with surgical resection. We also review the available literature regarding the presentation, diagnosis, and management of LAMN.

## Case presentation

**Figure 2:**
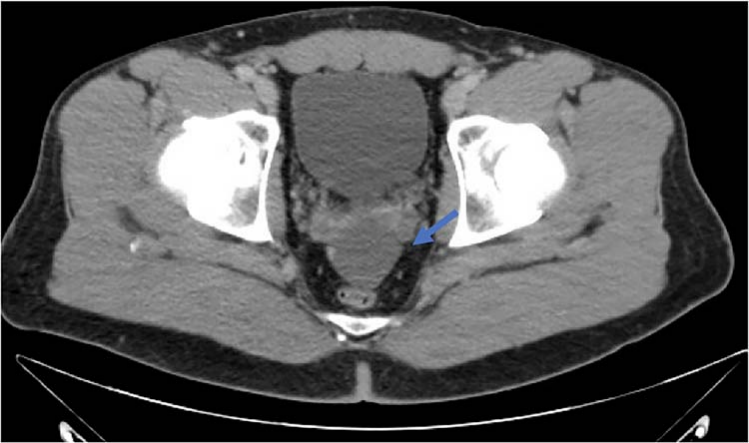
Fluid collection is present in the cul-de-sac that measures 5.8 × 4.8 × 2.9 cm (blue arrow).

An active 41-year-old male with no significant past medical, surgical, or significant family history presented to the emergency department for evaluation of right lower quadrant abdominal discomfort that started three days prior to arrival. He did not report any associated trauma or systemic signs of disease, including fever, chills, night sweats, or weight loss. On physical exam, the patient did not appear to be in any distress and was otherwise well-appearing. He had mild right lower quadrant tenderness on palpation without rebound, guarding, or peritoneal signs. Diagnostic studies included a contrast-enhanced CT scan of the abdomen and pelvis, which revealed two fluid collections in the region of the appendix and the cul-de-sac, measuring ~21 Hounsfield units, without evidence of active inflammation (Fig. 2). His labs were otherwise unremarkable. Given these findings, he was initially treated for perforated acute appendicitis with abscess. Antibiotics were started, and the decision was made to drain the fluid collection. Diagnostic laparoscopy or drainage of the fluid collection by interventional radiology was not pursued given the risk of potentially seeding a malignant process. Since the patient continued to have persistent symptoms and the differential diagnosis included lesions with malignant potential, the decision was made to proceed with exploratory laparotomy for both diagnostic and therapeutic purposes.

**Figure 3:**
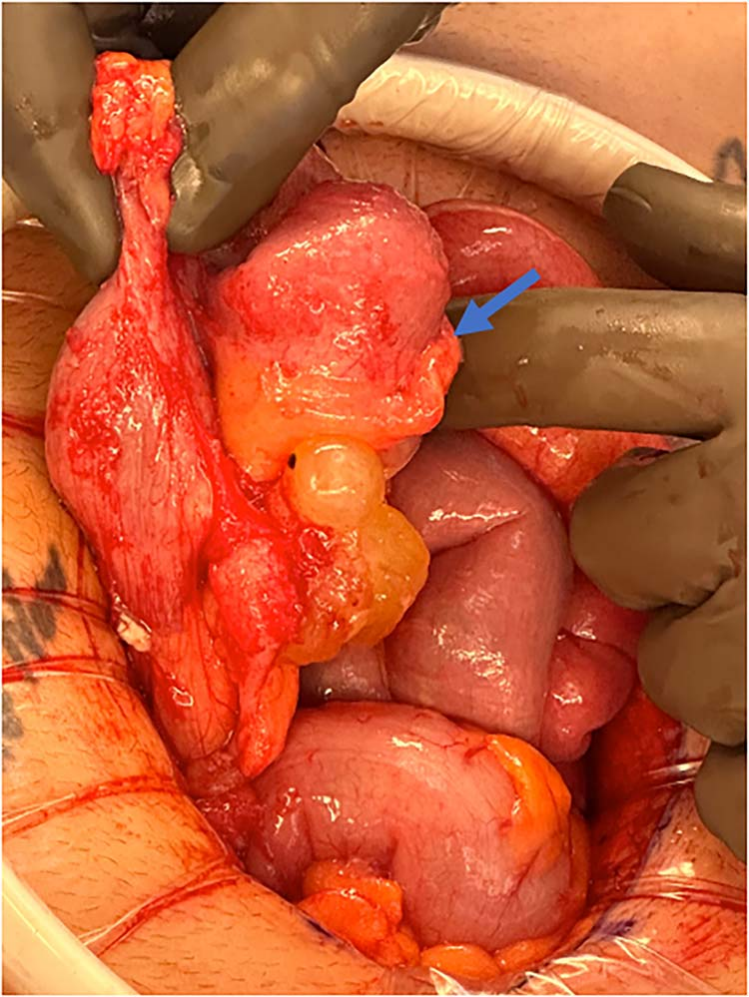
Perforated mucinous mass at the base of the appendix (blue arrow).

**Figure 4:**
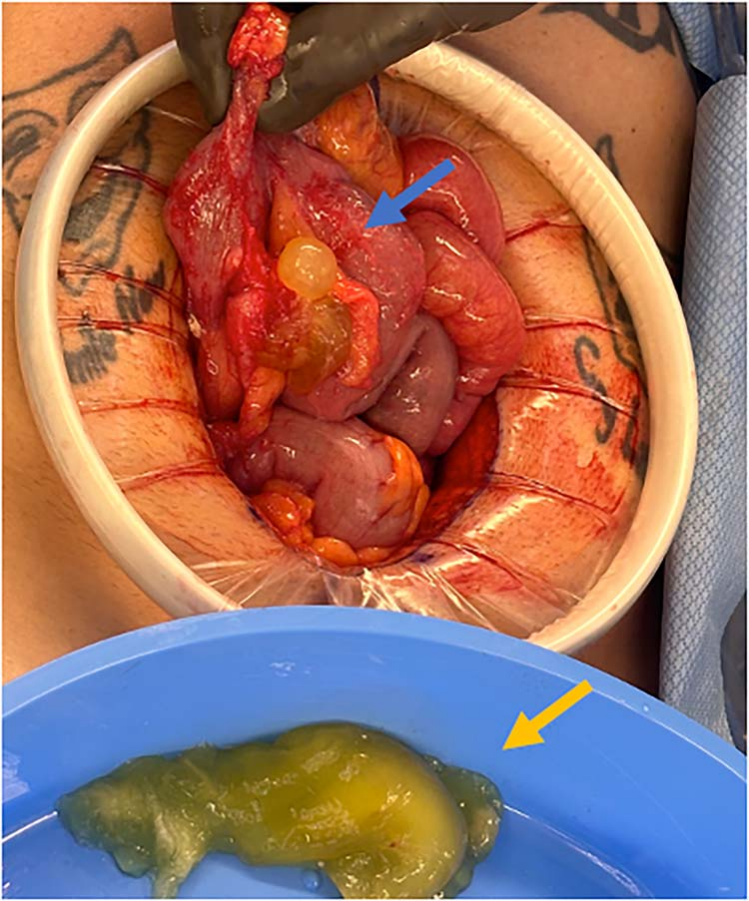
Perforated mucinous mass at the base of the appendix (blue arrow) and mucin collection at the region of the appendix (yellow arrow).

**Figure 5:**
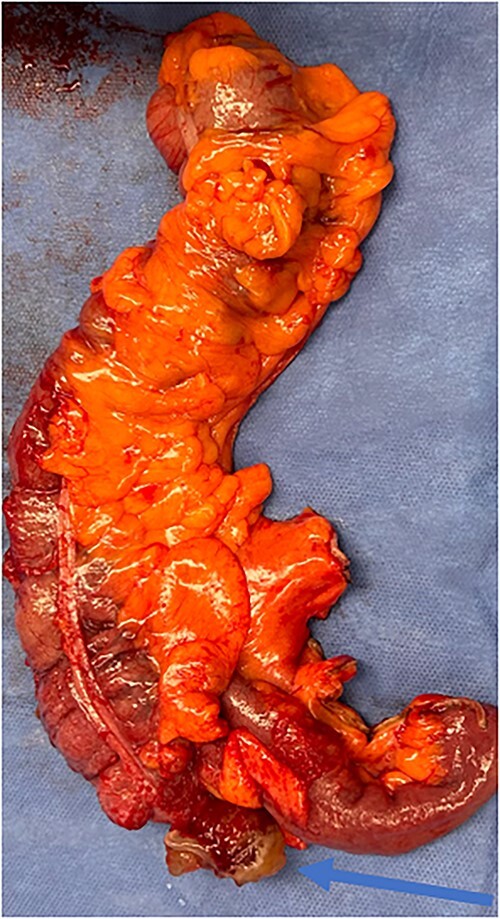
Specimen including terminal ileum, ascending colon containing the perforated mucinous mass at the base of the cecum (blue arrow).

An exploratory laparotomy with a midline incision was performed. Upon inspection of the abdominal cavity, there was no evidence of metastatic disease to the peritoneum, omentum, or liver. A perforated mucinous mass with mucinous collection was identified in the right lower quadrant by the appendix with an additional mucinous collection in the pelvis. Given the appendiceal and cecal involvement, the decision was made to perform a right hemicolectomy and primary anastomosis. Given that this was favored to be a malignant process intraoperatively, the decision was made to perform a right hemicolectomy over a limited ileocolic resection. Lymph nodes were also included due to the extent of the surgical resection. The specimens were sent to pathology. The abdomen was further inspected and subsequently closed (Figs 3–5). The patient tolerated the procedure well, was extubated in the operating room, and recovered in the post-anesthesia care unit in stable condition. The patient was observed postoperatively and was discharged home on postoperative Day 8. The duration of follow-up is 4 months without clinical concerns during follow-up. The final pathology of the specimens revealed LAMN with perforation and associated calcifications. The proximal and distal resection margins were free of neoplasia, and 42 lymph nodes were negative for tumor. There was presence of acellular mucin in the tissue sample, suggesting a favorable prognosis according to previous studies demonstrating that 96% of patients with acellular extra-appendiceal mucin were disease-free at 52 months [5]. Follow-up via surveillance magnetic resonance imaging (MRI) every 6 months, and colonoscopy were recommended. The patient was referred for blood testing, including the tumor marker carcinoembryonic antigen (CEA), which has been unremarkable.

## Discussion

LAMN is a rare entity present in <1% of appendectomies [1, 2], and diagnosis is difficult given its variable presentation, lack of specific clinical symptoms, and absence of a specific tumor marker [9]. In ~25%, patients are asymptomatic [1]. However, as we have seen in our patient, LAMN may also present as right lower quadrant pain as a result of the dilation of the mass, which may be mistaken for acute or chronic appendicitis. Several studies have sought to develop a surgical treatment algorithm based on the presentation and indications with no definitive conclusion given the rarity and variable presentation [9–12]. If the diagnosis of LAMN is missed, it may rupture and disseminate mucin and neoplastic cells into the peritoneal cavity, resulting in pseudomyxoma peritonei, which is an intraperitoneal accumulation of mucinous tumors and ascites, leading to high mortality [1–3, 13]. It is therefore crucial to diagnose these early and manage them appropriately. In this patient, the initial presentation and clinical suspicion were consistent with appendicitis with perforation. However, there was underlying caution that this may also be a malignant process. Although the presentation of LAMN may be variable and can mimic appendicitis, LAMN was not specifically suspected in this case given its rarity. We pursued exploratory laparotomy given the persistent symptoms and diagnostic uncertainty.

Imaging is an important adjunct in the diagnosis of LAMN. When not perforated, LAMN may have an ‘onionskin’ appearance on ultrasound. However, LAMN is most diagnosed with CT imaging with common features, including thin-walled low-density focus with smooth cystic walls, uniform intraluminal density, mostly with wall calcification, clear surrounding fat space, and intact cystic walls [12]. Alternatively, appendicitis may show blurred surrounding fat space, uneven density in the cavity, and thickening of the appendiceal wall [12]. Colonoscopy, if performed, may reveal a smooth mass originating from the opening of the appendix without visualization of the mucinous tissue [12]. MRI may be another test used to help diagnose LAMN, revealing a hyperintense appendix and bright mucin appearance on T2-weighted MRI [5]. Biopsy is typically not recommended given the risk of perforation and seeding [5]. In this patient, the CT scan findings revealed that there were two fluid collections in the region of the appendix and the cul-de-sac without the obvious findings of LAMN, including soft tissue thickening or wall irregularity [12].

In terms of surgical treatment, options may include appendectomy for limited disease. Right hemicolectomy, peritonectomy, and hyperthermic intraperitoneal chemotherapy (HIPEC) can also be considered for advanced disease [14]. When appendectomy is performed, LAMN and other suspicious lesions should be resected with confirmation of negative margins on pathology. Although lymphadenectomy is typically not required, lymph nodes may be present within the surgical specimen depending on the extent of resection [15, 16]. While laparoscopic surgery can be considered, laparoscopic dissection, grasping of the specimen, pneumoperitoneum, and transport of the specimen through the abdominal wall may contribute to the dissemination and progression of disease [17–19]. In this patient without clinical or radiographic signs or symptoms of an advanced process or systemic disease, upfront surgical exploration and resection was a reasonable option.

The current literature suggests that a conservative approach with routine imaging can be considered to monitor for recurrence [20]. For patients with advanced disease, such as pseudomyxoma peritonei, cytoreductive surgery, and HIPEC are standard treatments [21]. In this patient without evidence of advanced disease and negative resection margins, the conservative option with routine imaging was pursued.

While LAMN may present as a case of appendicitis, our findings represent an unusual presentation of right lower quadrant pain given that this was a healthy young male who presented without clinical or radiographic evidence of an acute inflammatory process. The diagnostic uncertainty of the presentation prompted initial treatment for appendicitis to prevent progression of disease; however, the persistent symptoms indicating failure of non-operative management and further clinical suspicion for malignancy prompted exploration. While a malignant process was suspected, LAMN was not specifically at the top of the differential since it is such a rare entity. If this patient were to be treated conservatively for appendicitis with antibiotics and/or a drain, the result may have been progression to advanced disease or potentially seeding of disease, respectively.

LAMN can progress to pseudomyxoma peritonei through perforation, and in this patient with persistent pain concerning for complicated appendicitis along with an underlying clinical suspicion for malignancy, surgical exploration may have prevented a potentially protracted clinical course. This condition can often be challenging to manage. Our case reports seek to contribute to the ongoing literature and provide a review of the current guidance.

## Conclusion

LAMN may present as acute onset or persistent right lower quadrant pain in a healthy patient. While we have noted that LAMN is typically discovered in females and in patients over 50 years of age, a high index of suspicion is warranted for all patients to prevent possible progression of disease. This condition can often be challenging to manage. Our case reports seek to contribute to the ongoing literature and provide a review of the current knowledge.

## Limitations

Limitations of our case report include our short-term follow-up at 4 months. A long-term follow-up would be beneficial in elucidating the sequelae of LAMN in our patient and associated outcomes. Further documented cases of rare presentations of LAMN may help provide further understanding of the nature of the disease and to bring awareness to the surgical and oncologic community.
